# Digital PCR Assays for Precise Quantification of CD19-CAR-T Cells after Treatment with Axicabtagene Ciloleucel

**DOI:** 10.1016/j.omtm.2019.12.018

**Published:** 2020-01-15

**Authors:** Boris Fehse, Anita Badbaran, Carolina Berger, Tanja Sonntag, Kristoffer Riecken, Maria Geffken, Nicolaus Kröger, Francis A. Ayuk

**Affiliations:** 1 Department of Stem Cell Transplantation, University Medical Center Hamburg-Eppendorf, Hamburg, Germany; 2 Research Department Cell and Gene Therapy at Department of Stem Cell Transplantation, University Medical Center Hamburg-Eppendorf, Hamburg, Germany; 3 Institute for Transfusion Medicine, University Medical Center Hamburg-Eppendorf, Hamburg, Germany

**Keywords:** Axicabtagene Ciloleucel, Axi-cel, Yescarta, Chimeric antigen receptor (CAR), digital PCR (dPCR), CAR monitoring

## Abstract

Treatment with axicabtagene ciloleucel (Axi-cel) CD19-CAR-T (chimeric antigen receptor T) cells has been approved for refractory/relapsed diffuse large B cell lymphoma (DLBCL) and primary mediastinal large B cell lymphoma (PMBCL). Because treatment success as well as side effects might depend on CAR-T cell expansion *in vivo*, we aimed at developing digital PCR (dPCR) assays for detection and quantification of CAR-T cells. To this end, we cloned and sequenced the complete cDNA of the CAR construct. We designed different combinations of primers and dual-labeled hydrolysis probes located in various CAR regions. Three combinations were successfully tested on CAR-positive and -negative cells in duplex reactions with a reference gene (REF) to concomitantly assess cell numbers. All assays demonstrated excellent specificity and reproducibility with neglectable inter-assay variations. For all three assays, almost perfect correlation between the two dPCRs (Axi-cel versus REF) was observed, and the limit of detection was one single CAR-transduced cell corresponding to a sensitivity of 0.01% for 100 ng genomic DNA. After cross-validation, we used one assay to monitor Axi-cel CAR-T numbers in patients. CAR-T expansion and contraction followed the expected kinetics with median peak value of 11.2 Axi-cel CAR-T cells/μL at 11.3 days (median). Clinically, we observed only two partial responses (PRs) in the five patients with CAR-T cell peak numbers below median, whereas four of the five patients with comparatively good expansion showed clinical responses (two complete responses [CRs] and two PRs) on day 30. In conclusion, we established a novel dPCR assay for the sensitive detection of transgenic CAR-T cells, which should be very useful in the context of Axi-cel treatment.

## Graphical Abstract

**Figure:**
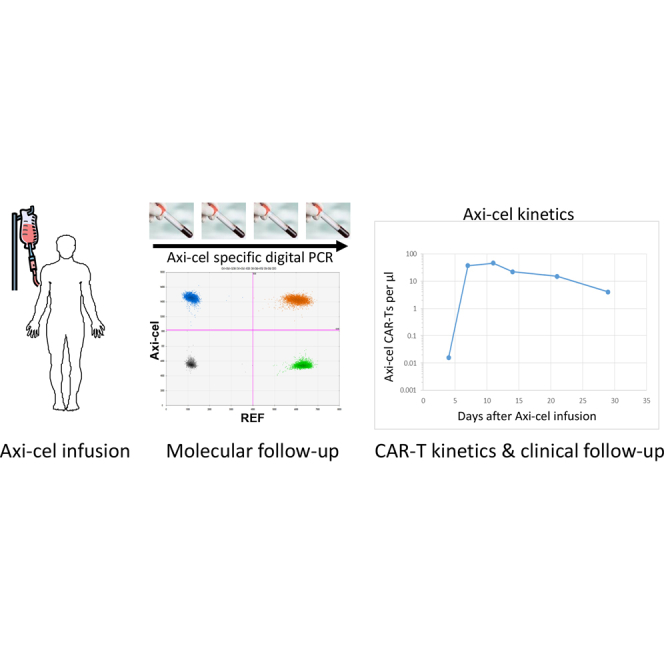

## Introduction

Chimeric antigen receptor T (CAR-T) cells have become a new treatment modality in hematology.^1^ Recently, two CD19-CAR-T cell products (tisagenlecleucel/Tisa-cel/Kymriah and axicabtagene ciloleucel/Axi-cel/Yescarta) have been licensed for the treatment of different B cell malignancies.^1^^,^^2^ CAR-T cell engraftment and expansion *in vivo* were shown to represent crucial parameters for efficacy of Axi-cel treatment.^3^^,^^4^ In particular, CAR-T cell peak concentrations and area under the curve in the first 28 days after Axi-cel infusion have been associated with long-term efficacy.^3^^,^^4^ Indeed, low initial CAR-T expansion seems to be associated with poorer outcome. However, additional data are necessary in order to adapt treatment protocols based on CAR-T engraftment. Unfortunately, diagnostic assays to quantitatively assess CAR-T cells *in vivo* are currently missing and thus are urgently needed.

Digital PCR (dPCR) represents an advancement of quantitative PCR, which is based on limiting-dilution and Poisson statistics.^5^^,^^6^ It is characterized by excellent sensitivity, specificity, and reproducibility, and is therefore considered an ideal tool for the quantitative determination of rare events, e.g., for MRD and chimerism diagnostics in hematology,^7^^,^^8^ but also the detection of gene-modified cells *in vivo*.^9^

At our center, 16 patients have been treated with Axi-cel so far. In order to assess engraftment, expansion, and persistence of CAR-T cells, we aimed at establishing a dPCR assay for precise quantification of gene-modified cells in clinical samples. We here describe the development, testing, and clinical application of this assay.

## Results

### Establishment and Testing of dPCR Assays

**Figure 1:**
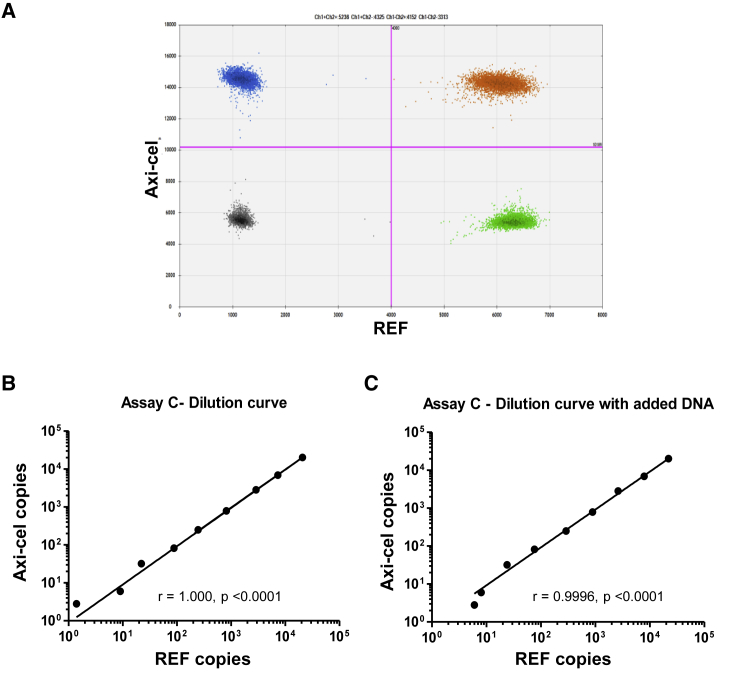
Digital PCR Assays Facilitate Sensitive and Accurate Quantification of Vector Copies (A) Perfect separation of single- and double-positive droplets in duplex PCR concomitantly detecting Axi-cel and REF sequences as exemplified for assay C. (B) Dilution series (100 ng → 10 pg) of genomic DNA isolated from T cells containing two REF and approximately two vector copies per cell demonstrate excellent correlation of both Axi-cel and REF-directed dPCRs. (C) The presence of additional genomic DNA (100 ng per probe) in the samples had no negative impact on sensitivity or correlation (right plot). Pearson coefficients (r) and corresponding p values are indicated. 10-pg samples corresponding to three REF and Axi-cel copies were consistently positive, indicating a limit of detection (LoD) of 1 copy for both dPCRs (considering Poisson distribution).

We first assessed dPCR performance for three primer/probe combinations (amplicons A, B, and C) on CAR-positive and -negative cells. As exemplarily illustrated for amplicon C, we observed ideal separation of negative and positive droplets (Figure 1A). Mixed DNA from 20 buffy coats as well as pre-infusion samples from patients were negative with the exclusion of one slightly positive PCR (0.07%) with amplicon A in a pre-infusion sample of patient #001; the sample was negative with both amplicons B and C.

To assess sensitivity and conformity, we performed duplex dPCRs on serial (half-log) dilutions of genomic DNA (gDNA) samples from one original product with a mean of approximately two vector copies per cell (i.e., both Axi-cel and REF [reference gene] were diploid) with all three assays resulting in almost perfect correlation of the two PCRs (Figure 1B). Notably, consistent positivity of the last (10-pg) samples (in all three assays) indicated a limit of detection (LoD) of 1 for both Axi-cel and REF. Indeed, 10 pg gDNA would be expected to contain a mean of three copies of any diploid gene or transgene. Considering Poisson distribution, the presence of a mean of three copies per tested volume unit would result in 95% of individual tests actually containing at least one copy, which in turn would be detectable by PCRs with an LoD of 1. At the same time, at the LoD level, independent Gaussian distribution of the two PCR targets (Axi-cel and REF) in individual samples would be expected to result in a loss of correlation and thus linearity between the two parameters. This is indeed the case as illustrated in Figures 1B and 1C. Furthermore, addition of each 100 ng of third-party gDNA (from healthy donors) to the samples of the dilution series did not impair sensitivity of the assay (Figure 1C).

### Assay Reproducibility

**Figure 2:**
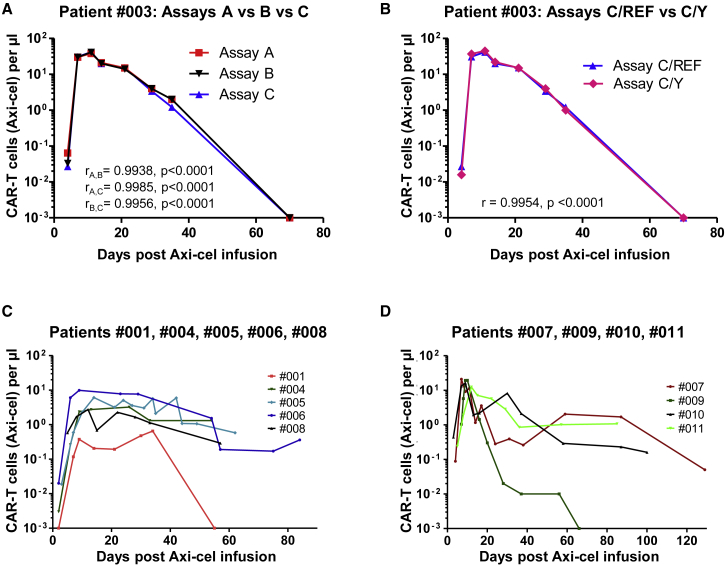
Axi-cel Kinetics in Patients (A) Excellent correlation of Axi-cel kinetics in patient #003 independently assessed using assays A, B, and C in duplex PCRs with REF. (B) For male patients (here: patient #003), replacement of the REF by a Y chromosome-specific dPCR in the duplex assay is possible without negative impact on results. (C and D) Individual patients show different engraftment and expansion kinetics of Axi-cel: (C) weak expanders (<11.2 Axi-cel CAR-T cells/μL) and (D) strong expanders (>11.2 CAR-positive Axi-cel T cells/μL). Negative values were set to 0.001%. LoD, limit of detection; r, Pearson coefficient; p, corresponding probability value.

To study reproducibility and potential inter-assay variations, we performed initial measurements in parallel using all three amplicons. We observed excellent coherence of the assays on tested Axi-cel products and patient samples (Table 1; Figure 2A). Assay C was selected for further use.

**Table 1:** Patient Characteristics and Data

	Patient Number									
	#001	#003	#004	#005	#006	#007	#008	#009	#010	#011
Age (at CAR infusion), years	57	57	78	68	55	47	24	40	44	79
Sex	male	male	male	male	female	female	female	male	male	female
Diagnosis	DLBCL	DLBCL	DLBCL	DLBCL	DLBCL	PMBCL	PMBCL	DLBCL	PMBCL	DLBCL
Axi-cel mVCNs (dPCR)^a^										
A		2.051	1.964	4.446	1.800	3.368	3.336			
B		2.077	1.972	4.378	1.790	3.340	3.411			
**C**	n.a.	**2.059**	**1.894**	**4.446**	**1.812**	**3.393**	**3.355**	3.3.81	4.536	3.313
Estimated transduction rate (%)^b^										
A		87.18	85.97	98.83	83.47	96.55	96.53			
B		87.47	86.08	98.75	83.30	96.46	96.70			
**C**^a^	n.a.	**87.24**	**84.95**	**98.83**	**83.67**	**96.64**	**96.51**	96.6	98	96.36
Estimated Axi-cel mVCNs per transduced cell^c^										
A		2.352	2.284	4.498	2.157	3.489	3.456			
B		2.374	2.291	4.434	2.149	3.462	3.527			
**C**^a^	n.a.	**2.360**	**2.229**	**4.498**	**2.166**	**3.511**	**3.477**	3	4.585	3.438
Axi-cel peak/μL (day)	1 (34)	42 (11)	3 (26)	6 (14)	10 (9)	21 (7)	3 (12)	19 (10)	16 (9)	12 (12)
CRS (grade)^d^	no	1	2	2	3	0	0	2	3	0
CRS treatment	no	no	no	no	Toci	no	no	Toci (2×)	Toci (2×)	no
ICANS (grade)^d^	no	1	4	1	1	0	0	0	2	0
ICANS treatment	no	no	no	no	no	no	no	no	Dexa (1×)	no
Best clinical response at day 30	PD	CR	PR	SD	PR	PR	SD	PR	PR	CR

CR, complete response; CRS, cytokine release syndrome; Dexa, dexamethasone; DLBCL, diffuse large B cell lymphoma; ICANS, immune effector cell-associated neurotoxicity syndrome; mVCN, mean vector copy number; n.a., not available; PD, progressive disease; PMBCL, primary mediastinal large B cell lymphoma; PR, partial response; SD, stable disease; Toci, tocilizumab.

a Data were obtained on Axi-cel infusion products. For the first seven patients, all three dPCR assays were used (A, B, and C). After finalizing evaluation, **assay C** was selected, and follow-up data were acquired with that assay only.

b Transduction rates were estimated using the Poisson formula based on the idealized assumption that all target cells were equally susceptible to transduction.^11^ Actual transduction rates might thus be lower.

c Axi-cel mVCNs per transduced cell were calculated based on the estimated transduction rates. Actual mVCNs per transduced cell might therefore be higher.

d CRS and ICANS grading were based on American Society for Transplantation and Cellular Therapy (ASTCT) Consensus criteria.^14^

We next studied reproducibility of assay C on individual samples. To this end, we selected five samples from different patients, which represented a broad range of Axi-cel concentrations (approximately 0.3–13 cells/μL). For each sample, we performed three new independent dPCR runs (Table 2, runs 2–4) to assess potential inter-assay diversity. In addition, all dPCRs were performed in triplicates within these additional runs to test intra-run conformity. As shown in Table 2, excellent reproducibility was found for all samples tested with the expected slightly higher variability at low cell levels due to normal distribution.

**Table 2:** Reproducibility of dPCR

No.	Measured Numbers of Axi-cel CAR-T Cells/μL										
	Run 1	Run 2			Run 3			Run 4			Mean
		A	B	C	A	B	C	A	B	C	
1	1.66	1.84	1.77	1.27	1.56	1.34	1.70	1.81	1.97	1.43	1.64
2	0.39	0.46	0.37	0.42	0.26	0.10	0.19	0.22	0.10	0.27	0.28
3	19.25	19.05	20.23	20.03	19.93	19.03	18.39	18.6	18.56	18.54	19.16
4	0.44	0.28	0.57	0.22	0.31	0.45	0.50	0.18	0.40	0.34	0.37
5	13.4	13.25	13.47	12.99	14.38	12.78	12.78	12.95	12.82	12.98	13.18

Run 1 was performed as the diagnostic assay. Runs 2–4 represent independent dPCRs, all carried out in triplicates, labeled A, B, and C.

For one male patient, dPCR analyses were repeated replacing the diploid REF gene for a haploid REF gene located at the Y chromosome^10^ with identical results (Figure 2B).

### Quantification of Axi-cel Vector Copies

Mean vector copy numbers (mVCNs) were first assessed in the Axi-cel products, using leftovers from the infusion bags. We found relatively high mVCNs in all samples with excellent inter-assay conformity (Table 1).

Because mVCNs in the cell product had been determined for all cells (including non-transduced ones), we used Poisson statistics to define the mVCNs per transduced cell (see Materials and Methods). This approach is based on the idealized assumption that all cells were equally susceptible to transduction; it might therefore overestimate transduction rates and underestimate mVCNs in actually transduced cells.^11^ However, it represents the best available approximation if gene transfer rates are unknown.

The calculated mVCNs per transduced cell (e.g., 2.36 for patient #003) were subsequently used to compute actual numbers of Axi-cel-positive cells using dPCR data for individual time points. For example, for patient #003, we measured 352 CAR and 8,620 REF signals at day 11 post Axi-cel infusion. The 8,620 REF signals correspond to 4,310 genome equivalents (or cells), because REF is a diploid gene. Accordingly, the 352 CAR signals correspond to 149 CAR-positive cells, because CAR-positive cells contained 2.36 vector copies in the mean. Together, an estimated 3.45% (149/4,310) of the white blood cells (WBCs) were CAR-T cells at day 11. Given his WBC count of 1.2 at this day, his CAR cells peaked at approximately 42/μL (Figures 2A and 2B). Calculations were accordingly adapted for MNCs used in subsequent analyses.

### Analysis of Patient Samples

During the initial method validation, patient samples were analyzed using the three dPCR assays in parallel, before we focused on assay C.

Because no product sample was available for patient #001, we assumed an mVCN per cell of 2 (corresponding to the lowest measured value in all products). Consequently, data obtained for this patient might overestimate actual numbers of Axi-cel cells in his blood. In any case, we saw very limited Axi-cel expansion and early loss of CAR-T cells (Figure 2C) corresponding to his clinical course of fast-progressing disease. Similar kinetics were found in patients #004, #005, #006, and #008 (two stable disease [SD] and two partial responses [PRs]; Figure 2C; Table 1). In contrast, patients #003 (Figures 2A and 2B), #007, #009, #010, and #011 (Figure 2D) showed the expected rapid CAR-T expansion with peaks above the median (11.2/μL) at days 7–12 accompanied by better clinical responses (two complete responses [CRs] and three PRs). Individual Axi-cel CAR-T kinetics are summarized in Figures 2C and 2D, and patient characteristics in Table 1.

## Discussion

Recent approval of the two CD19-CAR-T cell products (Tisa-cel and Axi-cel) for the treatment of relapsed/refractory B cell malignancies has underlined the huge potential of targeted cellular immunotherapies. Earlier clinical trials had identified efficient engraftment and expansion of CAR-T cells as a sine qua non for their efficacy,^3^^,^^4^ but the actual impact of engraftment kinetics on clinical efficacy of CAR-T cells and the risk for severe side effects such as cytokine release syndrome (CRS) and immune effector cell-associated neurotoxicity syndrome (ICANS) has remained largely obscure. These data are obviously essential in order to optimize treatment strategies in non-responders and patients with severe side effects. Therefore, the current lack of diagnostic assays to quantitatively assess CAR-T cells *in vivo* is quite surprising and needs to be overcome.

We have established dPCR-based assays combining excellent sensitivity, specificity, and reproducibility for follow-up diagnostics after Axi-cel treatment. All three assays target different regions of the CAR-vector construct and are therefore highly specific for Axi-cel products. At the end of the evaluation phase we chose one assay for further use. The LoD of 1 vector copy as established for this assay technically corresponds to a limit of quantification (LoQ) of 3; i.e., a sample containing a mean of three target copies in the test volume could be expected to become positive with a likelihood >95%.

In practical terms, if 100 ng gDNA (equivalent to approximately 15,000 cells or 30,000 haploid gene copies) will be tested, the given LoQ of 3 would translate into an actual sensitivity of 1 in 10,000 (3 in 30,000), or 0.01% (for any diploid gene). Because all tested Axi-Cel products contained ≥2 vector copies per cell, our routine testing of 120 ng DNA guaranteed a minimal sensitivity of 0.01%. Obviously, the sensitivity is even higher when the actual mean copy numbers of the CAR construct in transduced cells markedly excel 2, as in many of our cases. On the other hand, if gDNA isolated from whole blood containing large amounts of non-T cells would be used, the efficient sensitivity of the assay would be impaired. Therefore, we suggest using peripheral blood mononuclear cells (PBMCs) as starting material. Alternatively, the sensitivity might be increased by using larger gDNA amounts, but technical limitations might apply for a given dPCR system with regard to the maximally tolerable amount of gDNA. Also, the maximal number of quantifiable signals (using Poisson correction) is limited by the actual number of individual PCRs in a given dPCR device (e.g., approximately 20,000 droplets in the QX100/200). For the latter system, quantification at copy numbers >100,000 could be expected to be less precise. This would be relevant for the REF gene used in duplex PCRs as proposed here. Indeed, for a diploid gene the indicated upper limit of 100,000 copies would correspond to approximately 300 ng gDNA. Notably, using a Y chromosome-located haploid gene as reference allows doubling the DNA input in duplex dPCR. A further way to increase sensitivity is the use of sorted cell populations.

After thorough validation, we have applied one of the three assays for retrospective and prospective testing of patient samples. We performed diagnostic assays on gDNA isolated from PBMCs. In order to correctly determine the actual numbers of (Axi-cel) CAR-T cells, both the estimated vector copy number and the PBMC counts need to be taken into account. It cannot be excluded that mVCNs in transduced cells change over time. Although this is very unlikely in the early phase of polyclonal engraftment, it might become evident at later time points, when numbers of CAR-T cells (and thus also clones) will be reduced. However, the expected calculation error will be limited (e.g., a factor of 2 for a decrease in mVCNs from 4 to 2) and is therefore not expected to impact data significance, particularly at low CAR-T numbers, where presence is more relevant than numbers (e.g., 0.02 versus 0.01 Axi-cel CAR-T cells/μL).

The observed Axi-cel kinetics *in vivo* were in agreement with previously published results.^3^^,^^4^^,^^12^ Interestingly, we found a trend for an association between more pronounced expansion and clinical responses. Indeed, the five patients early reaching CAR-T levels above the median peak level of 11.2/μL showed good 30-day clinical responses (two CR, two PRs, and one SD), whereas treatment efficacy was less pronounced (one progressive disease [PD], two SD, and two PRs) in the five patients for whom CAR-T peaks were below the median (peaking at 10 CAR-T cells, patient 6 with PR also had good T cell expansion close to the median). However, these clinical observations are preliminary and still require validation in a much larger prospective patient cohort.

In conclusion, we suppose that our novel dPCR assay for the sensitive detection of CAR-positive T cells will be very useful for centers performing CAR-T cell therapy with Axi-cel.

## Materials and Methods

### Identification of Primer and Probes for dPCR

The complete cDNA sequence of the Axi-cel CAR construct was obtained by PCR and standard Sanger sequencing (eurofins Genomics, Ebersberg, Germany). PCR primers were selected by educated guess to bind the backbone of the γ-retroviral vector in front of the CAR’s cDNA (5′-ACCGCCCTCAAAGTAGACGG-3′) and behind the CAR’s cDNA in reverse orientation (5′-ACCTACAGGTGGGGTCTTTCA-3′). The amplified PCR fragment was then sequenced using the same forward primer. Based on the obtained sequence, an internal primer was designed to sequence the second half of the CAR’s cDNA (5′-CGGCCGCAATTGAAGTTATGT-3′). The full-length sequence was found to be identical to the one previously published by Kochenderfer et al.^13^

We used PrimerExpress_3.0.1 (Thermo Fischer) to design primers/probes for three different amplicons (A, B, C) located in different regions of the CAR construct. To minimize cross-reactivity with human genes, we put the two primers of each amplicon on different functional domains of the CAR. The final primer/probe combination (C) is available from Bio-Rad (Foster City, CA, USA) as an Expert Design Assay (catalog [Cat.] number dEXD45718942).

### gDNA and dPCR

gDNA was prepared from unseparated peripheral blood (PB) or PBMCs with QIA-Amp Blood Kit (QIAGEN, Hilden, Germany) following the manufacturer’s protocols.^7^ dPCR was carried out with the QX100 Droplet-Digital PCR System (Bio-Rad) as described previously.^7^ To assess numbers of analyzed genome equivalents and ensure sample quality, we concomitantly amplified CAR and REF sequences using differently labeled (FAM and HEX) probes. We used previously described target sequences as REF genes, namely, the diploid *hematopoietic cell kinase* (*HCK*) gene and the (male-specific) haploid *DFFRY* gene.^10^ Final concentrations of primers (900 nM) and probes (250 nM) followed the general Bio-Rad guidelines for dPCR. We typically analyzed 120 ng gDNA corresponding to approximately 18,000 diploid genomes (cells) per sample. A total of 5 U EcoRI (Thermo Fischer, Kandel, Germany) was added to the reaction mix that was incubated at room temperature for 5 min before starting the PCR. Data were analyzed with QuantaSoft_v1.7 software (Bio-Rad) including automatic Poisson correction.^7^

### Patients and Patient Material

Samples from 10 consecutive adult patients (CAR#001, CAR#003–#011) who received Axi-cel treatment between March and June 2019 were analyzed with written informed consent. The study was approved by the local Ethics Committee (#PV7081). Starting from the second Axi-cel patient (CAR#003), leftovers of Axi-cel products after infusion were included into analysis. Patient characteristics are summarized in Table 1.

PBMCs were isolated from patient blood (or bone marrow) by Ficoll gradient centrifugation using SepMate (STEMCELL Technologies, Cologne, Germany) following the manufacturer’s instructions. To calculate PBMC numbers in a given sample, we determined differential blood counts using standard automated blood cell counters (Advia 2120i [Siemens, Erlangen, Germany], ABX Pentra XL80 [Horiba, Irvine, CA, USA]). PBMCs were determined as WBCs minus granulocytes (absolute neutrophil count, ANC) and/or lymphocytes + monocytes. At low WBC counts, blood cell populations were counted manually.

### Calculations and Statistics

To assess mVCNs in a given Axi-cel product, we performed duplex dPCRs with Axi-Cel and REF primers, and copy numbers were calculated for the two amplicons using QuantaSoft_v.1.7 software. Whereas REF copy numbers provided information on the absolute numbers of alleles (and thus cells) tested, the Axi-cel dPCR determined the absolute number of vector copies in this very sample. To define the actual mVCNs per transduced cells, it had to be taken in account that the cellular product contained both transduced and non-transduced cells. Provided that all cells are equally susceptible to transduction by the used retroviral vector, Poisson statistics can be applied to determine the transduction rate based on the measured vector copy number.^11^ Under this assumption, a mean copy number of, for example, 2 in an Axi-cel cell product corresponds to a transduction rate of 86.5%.^11^ Consequently, in such case, transduced cells contain a mean of 2.31 (2/0.865) copies.

Correlation coefficients were determined using two-tailed Pearson statistics with a confidence interval of 95%. Statistical analyses were made with GraphPad Prism (San Diego, CA, USA).

## Author Contributions

B.F. conceived the study and wrote the manuscript. A.B. designed, performed, and analyzed dPCRs. C.B. processed patient samples, and compiled and analyzed data. T.S. processed patient samples. K.R. cloned and sequenced the Axi-cel construct. M.G., N.K., and F.A.A. treated patients and provided clinical data. All authors reviewed and edited the manuscript.

## Conflicts of Interest

The dPCR assay described in this work has been made available as an “Expert Design Assay” based on agreement with Bio-Rad. B.F., A.B., C.B., and K.R. would profit from potential future commercialization of the assay. The other authors declare no competing interests.
